# The Interactions of Media Use, Obesity, and Suboptimal Health Status: A Nationwide Time-Trend Study in China

**DOI:** 10.3390/ijerph182413214

**Published:** 2021-12-15

**Authors:** Qinliang Liu, Xiaojing Li

**Affiliations:** School of Media & Communication, Shanghai Jiao Tong University, Shanghai 200240, China; liuql201807@sjtu.edu.cn

**Keywords:** media use, obesity, suboptimal health status, time trend, China

## Abstract

Obesity and suboptimal health status (SHS) have been global public health concerns in recent decades. A growing number of works have explored the relationships between media use and obesity, as well as SHS. This study aimed to examine the time trend of the associations between media use (including traditional media and new media) and obesity, as well as SHS. The data were derived from three national random samples of the Chinese General Social Survey (CGSS), which was separately conducted in 2013, 2015, and 2017. In total, 34,468 respondents were included in this study, consisting of 16,624 males and 17,844 females, and the average age was 49.95 years old (*SD* = 16.72). It found that broadcast use and television use were positively associated with obesity and showed an increasing trend over time. Cellphone use emerged as a risk factor for obesity in 2017 and showed an increasing trend. By contrast, newspaper use, television use, and internet use were negatively associated with SHS, and television use showed a decreasing trend in the association with SHS, while internet and newspaper use showed an increasing trend. In conclusion, media use was positively associated with obesity while negatively associated with SHS. It showed a decreasing trend in the associations between traditional media use and obesity, while revealing an increasing trend in the associations between new media use and obesity, as well as SHS. The practical implications of the findings are discussed.

## 1. Introduction

Over the recent decades, communication technology has changed dramatically. A variety of new media, including the internet, computers, cellphones, and social media have rapidly come to coexist with traditional media (e.g., television, newspaper, and broadcast media), which dominate individual’s leisure activities and spare time [1]. Media use has also been integrated into daily life to communicate with peers and maintain social relationships [2], search for or share information, and have fun or entertainment [3]. Despite the ease and frequency of media use having changed radically in recent years, these changes did not always bring positive impacts on health status, but did bring negative consequences [4]. In other words, media use is a two-sided coin that may improve one’s health status, but also could result in harmful health outcomes. Widespread and prolonged media use has contributed to the increasing and ongoing debate on its impacts on physical and psychological health, such as obesity or overweight, and the symptoms of suboptimal health status (SHS).

### 1.1. Media Use and Obesity

Obesity has been a global public health concern which has caused various health problems and social burden [5,6]. Recent work has indicated that obesity has become widespread in adolescents as well as in young and older adults [7,8], with an increasing trend in recent decades [9]. The rapid rise of obesity suggests that environmental factors along with biological disposition might be responsible for people’s weight gain [10]. Among the environmental factors, ubiquitous information communication technology and pervasive media use might account for the widespread obesity [11]. One of the possible explanations lies in the fact that electronic media use or screen media use have displaced time for physical activities or outdoor sports. The more or longer one uses such media, the more likely s/he is to become obese [12,13]. A large body of studies have also examined the associations between media use and obesity or overweight [14,15,16]. The scope of these studies has involved almost all media types, such as television [17], video games [18], internet and computer [19], social media [20], and digital media [21]. The research outcomes have demonstrated that media use has caused a decrease in physical activity and an increase in sedentary behaviors, which has resulted in the risk of obesity [22,23].

### 1.2. Media Use and Suboptimal Health Status

SHS is an intermediate state between health and disease conditions, characterized by declines in vitality, physiological function, and the capacity for adaptation [24]. It includes symptoms of fatigue, non-specific pain, dizziness, anxiety, depression, and functional system disorders [25]. In recent years, more and more people are in SHS. SHS has become a significant public health concern worldwide due to its potential risk for chronic diseases across multiple populations, such as type-II diabetes mellitus, cardiovascular and stroke [26,27].

According to the “biological-psychological-social” medical model, an individual’s health status is dictated by his/her biological, psychological, and social factors. Thus, SHS is usually measured or assessed by disturbance symptoms in terms of physiological, psychological, and sociological characteristics, namely physical SHS (PS), mental SHS (MS), and social SHS (SS) [28]. Given that SHS is a medically undiagnosed and functional somatic syndrome, it has been attributed to various factors. Existing studies have revealed that lifestyle and environmental factors were the critical predictors of SHS, such as sleep quality, physical exercise, unhealthy diet, smoking, alcohol drinking, adverse life events, and family living status [29]. In addition, the prevalence of SHS varies among different populations. Many demographic variables, such as age, gender, education, occupation, geographical region, and economic status, were associated with SHS [30].

As a critical predictor of SHS, screen-based media (SBM) has occupied a considerable portion of young peoples’ discretionary leisure time [3]. The overuse of electronic devices might be one of the underlying causes of SHS [31]. However, the results related to SHS were mixed. According to the social causation hypothesis, unreasonable media use could induce health problems since excessive or problematic media use increased the risk of poor sleep quality, obesity, lower life satisfaction, and anxiety [32,33]. In addition, social media provided a natural platform for social comparison, which led to more mental health disorders [34]. For instance, more social media use was proved to be positively correlated with weight dissatisfaction and poor body image, which resulted in lower self-esteem and higher depressive symptoms [35]. Those active on Facebook tended to score higher in terms of poor health status than those who used other social media platforms [2].

However, others have argued that individuals could significantly benefit from media use, whether in terms of physical health or psychological health. The social compensation hypothesis interprets that media use can compensate for what individuals lack in real life, including social resources and support [36]. Primarily, individuals view online social media as an attractive way to attain social support and social engagement which they lack offline [37]. These online media are easy to access and anonymous, providing users with a sense of connectedness and reducing feelings of isolation [38]. The new media enable individuals and communities to acquire and maintain social capital. All these benefits have a positive effect on mental health [39]. Besides, electronic or digital media have become the most popular platform for accessing health information [40]. They play critical roles in changing one’s attitudes toward health behaviors and persuading individuals to participate in health protection [41]. Furthermore, health-related media use was positively linked to health literacy [42], as well as eHealth literacy [43], which in turn influenced health behaviors and contributed to physical health [44].

### 1.3. The Current Study

Although prior studies have provided valuable knowledge to understand the relationships between media use, obesity and SHS [32,45,46,47], several limitations still exist in the body of scholarship. Firstly, most studies used cross-sectional data and demonstrated a concurrent relationship between media use and obesity or SHS. They could not demonstrate any causal effect over time [48]. Secondly, existing studies preferred to focus on a single type of media use to explore its associations with obesity or SHS, such as television, internet or social media, lacking a comparative perspective among different media [49,50,51]. Besides, most have focused on new media, ignoring traditional media which are still relevant, especially for certain age groups. Thirdly, most studies were executed in developed countries. However, developing countries like China are experiencing severe obesity and SHS problems [52] and encountering dramatic changes in media technologies in the 21st century.

Therefore, to gain a comprehensive and longitudinal view of the influence of media use, it is necessary to test the time trend of the supposed causality between media use and health status, as well as to consider the disparities between different media uses. The current study aims to reveal the time changes in the associations of media use with obesity and SHS from 2013 to 2017. Further, to compare the effects of different media use, we divided the media genres into traditional media (newspaper, magazine, broadcast, and television) and new media (internet and cellphone). Hence, the specific research questions were proposed:

- What are time trends in the associations of media use with obesity (RQ1) and SHS (RQ2) in the period 2013 to 2017?
- What are the differences in time trends between new media use and traditional media use, separately, in their associations with obesity (RQ3) and SHS (RQ4)?

## 2. Materials and Methods

### 2.1. Data Collection

The data was drawn from the Chinese General Social Survey (CGSS) in 2013, 2015, and 2017. The CGSS is a nationally presentative and continuous social survey project, implemented on the Chinese mainland by the Renmin University of China and Hong Kong University of Science and Technology annually since 2003. It has been broadly considered as the Chinese counterpart of the General Social Survey (GSS) in the United States. Using a stratified multi-stage probability proportionate to size (PPS) sampling method, samples were drawn from households in all 31 provincial units in Mainland China with three levels in each sampling frame: county or district, community, and household. In a selected county (district), four community-level units (neighborhood committees or village committees) were randomly selected. In a selected community-level unit, 25 households were selected, and in each selected household one adult was randomly selected for the survey [53,54]. CGSS is a household survey aimed at adults, and adolescents under 18 years were excluded. All the questionnaires were completed by face-to-face interview with the help of interviewers. 12,000, 10,968, and 12,582 sample cases were collected from 480 villages and urban neighborhood communities from 140 residential districts in 2013, 2015, and 2017, respectively. After excluding the missing answers, e.g., one participant who responded to the options “I don’t know” or declined to answer, or in the case of an incomplete questionnaire, then the case was removed from the database. Finally, 11,263, 10,383, and 12,367 respondents were retained in the samples for 2013, 2015, and 2017, respectively. All the samples were pooled as the analysis database.

### 2.2. Measures

#### 2.2.1. Media Use

Media use was measured by the following question: “In the past year, how often did you use the following media?” Media types consisted of newspaper, magazine, broadcast, television, internet, and cellphone. The answers were independently rated as “never”, “seldom”, “sometimes”, “often”, and “always”.

#### 2.2.2. Obesity

Obesity was indicated by body mass index (BMI) that was calculated as weight/height^2^. According to obesity criteria, the WHO defined overweight in adults as a BMI of 25.0–29.9 kg/m^2^ and obesity as a BMI of 30.0 kg/m^2^ or higher. However, accumulated evidence has consistently supported the use of lower BMI cutoffs in Chinese than those in whites [55,56,57], and the Working Group on Obesity in Asia also recommended BMI cutoffs of 24·0 kg/m^2^ to define overweight and 28·0 kg/m^2^ to define obesity [58]. As proposed by Zhou (2002) [59] for Chinese adults, the standard weight was defined as a BMI < 24 kg/m^2^, overweight as a BMI of 24–27.9 kg/m^2^, and obesity as a BMI ≥ 28 kg/m^2^. Consequently, each respondent was characterized as obese or not by the cut-off value of 28. The attribute of obesity was marked as “yes” or “no”.

#### 2.2.3. SHS

SHS was measured by three questions with self-defined items. According to the “biological-psychological-social” medical model, SHS was defined as physical SHS (PS), mental SHS (MS), and social SHS (SS) [28]. We selected the three most related questions responding to the three facets. The first two questions were: “In the past four weeks, what is the frequency of health problems affecting your work and other daily lives?” and “In the past four weeks, how often did you feel depressed?” The answers were rated as “never or seldom”, “occasionally”, “sometimes”, “often”, and “always”. The third question was, “What is your current health status?” The answers were rated from 1 to 5, as “excellent”, “good”, “fair”, “poor”, and “very poor”. All the answers were inversely coded. 

According to prior studies’ outcomes for Cronbach [60] and Bland & Altman [61], Cronbach’s alpha was used to investigate the internal consistency of the questionnaire, and the alpha should be more than 0.7 to 0.8 for research purposes and at least be 0.90 for clinical purposes. In this study, the Cronbach’s alpha of SHS was 0.774 (α = 0.774), indicating a better internal consistency for the constructed measurement items, which enabled SHS to be measured reasonably. Finally, the summed score of the three questions was marked as the measurement of SHS, and a higher score indicated a more severe SHS.

#### 2.2.4. Covariates

Covariates included sociodemographic characteristics, such as gender (male/female), age (years old), educational level (illiterate/primary school/middle school/high school/college or bachelor’s degree and above), and physical activity (inversely coded as “never”, “several times/year”, “several times/month”, “several times/week”, and “every day”). Hukou (rural/urban) was included to control for the unobserved geographic characteristics of the respondents’ residential location. In China, people are mainly divided into two groups (agricultural hukou holders and non-agricultural hukou holders) based on their birthplace and lineage. Generally, people with agricultural hukou live in rural areas with disadvantages in terms of the economy, education, medical, housing, and other social resources. By contrast, non-agricultural hukou holders who live in urban areas with more public and social resources have a high quality of life. So agricultural hukou and non-agricultural hukou are also called rural hukou and urban hukou [62]. Obesity was included in the regression analysis of the association between media use and SHS, coded as “yes” or “no”.

### 2.3. Statistical Analysis

Descriptive statistics were employed to describe the characteristics of sociodemographic indicators, media use, rate of obesity, and SHS. One-way ANOVA was used to analyze the P-trend of each variable by period. Logistic regression models were applied to estimate the associations between media use and obesity separately in 2013, 2015, and 2017. The analysis results were presented as odds ratios (ORs) with 95% confidence intervals (CIs), with all covariates being controlled. Multiple linear regression models were applied to estimate the associations between media use and SHS separately in 2013, 2015, and 2017. The results were presented as Beta and SE (standard Error) and controlled all covariates. To test whether the associations between media use and obesity as well as SHS varied over time, the cross-sections were pooled pairwise (e.g., 2013–2015, 2015–2017, and 2013–2017), and dummy-coded as an independent variable separately. For example, in the period of 2013–2015, 2013 was coded “0” and 2015 was coded “1”. Interactions of media type with the pairwise year were included separately in the adjusted models to determine whether the association changed significantly over the study periods [63,64]. All statistical analyses were conducted via SPSS 25.0. *p*-values were two-sided, and a value of less than 0.05 was considered statistically significant.

## 3. Results

### 3.1. Characteristics of Demographics, Media Use, Rate of Obesity, and SHS

Table 1 shows the characteristics for demographics, media use, rate of obesity, and SHS. In total, 34,468 respondents were included in this study, comprising 16,624 males and 17,844 females, and the average age was 49.95 years old (*SD* = 16.72). More than half of the respondents had less educational experience (64.08%) and were rural Hukou (58.98%), and all the respondents did physical activities (Mean = 2.34, *SD* = 1.52) at a relatively low level. As for the frequency of media use, television was rated most among the six media types, while it showed a decline and downward trend from 2013 to 2017, as well as newspapers, magazines, and broadcast. However, internet and cellphone use had an increasing tendency during the same time period. In addition, the prevalence of obesity increased from 5.7% to 7.6%, and the score of SHS increased from 6.39 (*SD* = 2.61) to 6.84 (*SD* = 2.65) during 2013 to 2017. All changes between different years were statistically significant.

### 3.2. Associations between Media Use and Obesity, and Time Changes

Table 2 shows the outcomes for logistic regression analysis on the associations between media use and obesity after controlling for age, gender, education, and physical activity. The associations varied by each time point. In 2013, television use was significantly and positively associated with higher odds of obesity, while magazine use was associated with decreasing odds of obesity. Broadcast and television were the significant predictors of obesity in 2015, whereas cellphone use emerged as a new predictor of obesity in 2017. Internet use was not significantly associated with obesity at any time point. These results answered RQ1.

As for the time trend of the association of media use with obesity (see Table 3), the significant interaction term indicates a time trend in pairwise time, but whether it is a decreasing or increasing trend depended on the changes of the predictive effect of media use on obesity during that period. Therefore, newspaper use showed a decreasing trend in the association with obesity since the positively predictive effect declined from 2013 to 2017. Magazine use negatively predicted obesity in 2013, but the predictive effect declined significantly during 2013–2015 and 2015–2017, which showed decreasing association trends over those periods. Broadcast use exhibited a decreasing time trend in the association with obesity during 2015–2017, because the positively predictive effect declined in that period. The positively predictive effect of television use on obesity declined from 2013 to 2017, but these changes were not significant. Therefore, there was no time trend in the association between television use and obesity. Cellphone use positively predicted obesity in 2017 and the predictive effect increased significantly during 2015–2017, thus the association between cellphone use and obesity showed an increasing trend in that period. These results answered RQ3.

### 3.3. Associations between Media Use and SHS and Time Changes

Table 4 shows the outcomes of linear regression analysis on the associations between media use and SHS after controlling for age, gender, education, physical activity, and obesity. Compared to obesity, the findings were somewhat different; excluding broadcast, the use of newspaper, television, and internet were negatively and significantly associated with SHS, separately in 2013, 2015, and 2017. Broadcast use was positively and significantly associated with SHS only in 2013. Magazine use was a significant predictor of SHS in 2017. Cellphone use was not a significant predictor of SHS at any time point. Thus, RQ2 was answered.

As for the time trend analysis of the association of media use and SHS (see Table 5), compared with 2013 and 2015 the negatively predictive effect of newspaper use on SHS increased significantly in 2017, thus the association of newspaper use and SHS had increasing trends both in 2015–2017 and 2013–2017. On the contrary, the positively predictive effect of broadcast use on SHS declined significantly during 2013–2015 and 2013–2017, so it presented a decreasing trend in the association and SHS, and the trend was more significant during 2013–2017. In addition, the negatively predictive effect of television use on SHS declined rapidly from 2013 to 2017, and all the changes were significant among these periods, which indicated decreasing trends in the associations between television use and SHS. By contrast, internet use negatively predicted SHS in 2015 and the predictive effect increased significantly in 2013–2015 and 2015–2017, thus the association between internet use and SHS showed an increasing trend in 2015–2017 and 2013–2017. In addition, magazine and cellphone use showed no significant time trend. These outcomes answered RQ4.

## 4. Discussion

Obesity and SHS have been worldwide health issues as living environments and life patterns have changed rapidly in the past decade [29,65]. The relationships between media use and obesity as well as SHS have been well documented in previous work [35,66,67]. This study aimed to better understand the media use profiles of Chinese adults and the changes in relationships between media use and obesity as well as SHS from a time trend perspective, based on a longitudinal national sample conducted among Chinese adults in 2013, 2015, and 2017.

It revealed that the profiles of media use changed significantly in China from 2013 to 2017, and the changes varied depending on different media types. Traditional media, such as newspapers, magazines, broadcast media and television use, presented a decreasing trend, and newspapers and television declined more significantly. On the contrary, the use frequency of new media (i.e., internet and cellphone) maintained a significantly increasing trend from 2013 to 2017, and internet use increased more than cellphone use. These trends were consistent with the dramatic progress in communication technologies and the rapid growth in Chinese internet users. According to the 34–39th Statistical Report on Internet Development in China, the number of Chinese internet users, internet penetration rate, and cellphone users had increased by 22.2%, 10.9%, and 41.7%, respectively, from 2013 to 2017 [68,69]. However, the opposite development trends of traditional media use and new media use did not mean that new media (i.e., internet and cellphone) had totally displaced the role of traditional media (i.e., newspapers, magazines, broadcast, and television) in China. Conversely, as our study revealed, television was still more popular than other media types among Chinese adults during the period to 2017. Internet was ranked as the second primary media use, behind television use. The possible explanation is that most of the participants had an older age, and may be prone to use traditional media (i.e., television) more than new media (i.e., Internet or cellphone) due to their relatively low level of media literacy or digital skills [70]. In addition, more than half of them were from rural areas, and may have a lower level of education and household economy, which reduces their chances of internet use [71].

Consistent with the time trend of new media use, both the prevalence of obesity and the magnitude of SHS increased significantly from 2013 to 2017, indicating that a higher proportion of Chinese adults suffered from overweight and health problems during that period. These findings were in line with the latest research findings that the prevalence of Chinese obesity changed from 3.1% (2.5–3.7) in 2004 to 8.1% (7.6–8.7) in 2018 [72], and more than half of Chinese adults showed SHS symptoms [24]. Obesity and SHS have become serious public health issues in China, but most current studies attribute the cause to economic developments, socio-cultural norms, food systems, and environment [73]. This study provided useful evidence for the assumption that media use was associated with obesity and SHS and found a significant time trend in these associations from 2013 to 2017.

Broadcast, television, and cellphone use were positively associated with obesity at different time points. Magazine use was negatively associated with obesity in 2013 while the association disappeared in 2015 and 2017. These findings suggested that both traditional and new media use might be risk factors for obesity. There was a significant time trend in the associations between media use and obesity in the study periods, and traditional media and new media demonstrated totally opposite tendencies. Traditional media use (i.e., magazine, broadcast, and television) showed a declining tendency in association with obesity from 2013 to 2017, while new media use (i.e., cellphone) showed an increasing tendency during that period. Cellphone use was increasingly important in predicting obesity and this trend was more significant in 2017 (see Table 2). The underlying mechanism of how media use influences obesity perhaps lies in the fact that media use increases sedentary possibilities and decreases physical activities.

Cellphones are usually characterized by multiple functions and are easy to use for all age groups compared with traditional media. Previous studies also found that cellphone did indeed occupy a large amount of leisure time and even led to cellphone addiction (extensive use or overuse), which brought about obesity or other health problems [74,75]. Furthermore, cellphone use was the only significant predictor of obesity in 2017, emphasizing that the cellphone had displaced traditional media and had the leading risk factor of obesity among the six media types in 2017. Nevertheless, it is worth mentioning that television use showed a decreasing tendency in associations with obesity from 2013 to 2017, while the time trend was not significant. In other words, television use was a stable predictor of obesity among the six media types over the study period. Therefore, television may be a potential risk factor for obesity in the future years.

In the existing literature, media use was demonstrated as a beneficial factor for SHS since it provided health information resources, social engagement, and social support [76]. In this study, newspaper use, television use, and internet use were negatively associated with SHS at each time point. Meanwhile, there were significant trends in the associations of media use with SHS, and the trends differed between media types.

Television showed a rapidly decreasing trend in the association with SHS from 2013 to 2017, while internet use showed a significant increasing trend. Further, the associations between internet use and SHS were stronger than the associations between television use and SHS in 2015 and 2017, indicating that internet use has displaced television and become the strongest predictor of SHS since 2015. The possible explanation is that the internet has been the leading platform and carrier of health information and has become more popular for health information access than television in the digital age [40]. Free access to vast online health information sources has created opportunities for empowerment, information exchange, and engagement in health-promoting behaviors [77]. In addition, online activities could promote well-being by reducing loneliness and facilitating social engagement [78], which might contribute to fewer SHS symptoms. Therefore, the internet should attach great importance to public health promotion in daily life. However, we should not overlook the role of traditional media in health communication, especially for television and newspapers, which were significant predictors of SHS in our study. Traditional media are still valuable resources for providing health-related information for people of low socio-economic status [79].

## 5. Limitations and Future Studies

The current study is one of the few that looked at time changes in the associations of different media use with obesity and SHS. Although substantial outcomes were seen when interpreting the results, the following limitations should be considered. Firstly, the repeated cross-sectional data did not allow us to draw causal inferences about the relationships between media use and obesity or SHS. Thus, a panel study and follow-up data are recommended to assess the causal effect of media use on obesity or SHS in future. Secondly, based on second-hand data, the measures of SHS were on a self-defined scale with limited items, which failed to measure SHS comprehensively and precisely. Although we made efforts to better match the three facets of SHS (i.e., physical SHS, mental SHS, and social SHS), the measures were single item and participants may have responded to the question more subjectively in terms of self-rated physical or psychological health, which might underestimate or overestimate the accurate SHS level. Thus, our findings for SHS need to be validated in future studies and a more comprehensive measure of SHS, such as the Suboptimal Health Status Questionnaire-25 (SHSQ-25) [80], should be employed. Thirdly, as the outcomes indicated, most of the respondents were middle-aged or older adults, whose media use status differs from that of young adults or adolescents, or conversely. It was unclear whether the findings could be expanded to adolescents or young adults. Therefore, future studies on time trends of media use and health status should cover more age groups and compare adolescents and older adults.

## 6. Conclusions

Based on three nationally representative samples of CGSS from 2013 to 2017 we tested the time trend of media use and the associations with obesity and SHS. We considered the differences between traditional media use and new media use. The results confirmed significant time trends between media use and obesity as well as SHS from 2013 to 2017. Furthermore, our study highlighted that the trend differences between traditional media and new media use were significant. Namely, traditional media use showed decreasing trends, while new media displayed an increasing trend. These findings contribute to a better understanding of how changing media use is impacting adults’ health status.

## Figures and Tables

**Table 1:** Descriptive characteristics of demographics, media use, obesity, and SHS in 2013, 2015, and 2017.

	Total (*N* = 34,468)		2013 (*N* = 11,263)		2015 (*N* = 10,838)		2017 (*N* = 12,367)		*P* for Time Change
	*M*/*N*	*SD*/*%*	*M*/*N*	*SD*/*%*	*M*/*N*	*SD*/*%*	*M*/*N*	*SD*/*%*	
Male	16624	48.23%	5685	50.48%	5078	46.85%	5861	47.39%	
Female	17844	51.77%	5578	49.52%	5760	53.15%	6506	52.61%	
Age	49.95	16.72	48.56	16.37	50.32	16.88	50.90	16.80	
Hukou									
Rural	20297	58.89%	6739	59.83%	6862	63.31%	6696	54.14%	
Urban	14171	41.11%	4524	40.17%	3976	36.69%	5671	45.86%	
Education									
Illiterate	22087	64.08%	7265	64.50%	7114	65.64%	7708	62.32%	
Primary	6312	18.31%	2154	19.12%	1953	18.02%	2205	17.82%	
Junior high Middle school	2730	7.92%	915	8.12%	781	7.21%	1034	8.36%	
High school	2944	8.54%	837	7.43%	874	8.06%	1233	9.97%	
College or Bachelor and above	395	1.15%	92	0.82%	116	1.07%	187	1.51%	
Physical activity	2.35	1.52	2.07	1.38	2.46	1.53	2.50	1.60	
Newspaper	1.92	1.16	2.10	1.22	1.90	1.12	1.77	1.11	1–2, 1–3, 2–3
Magazine	1.71	0.95	1.83	1.01	1.72	0.94	1.61	0.90	1–2, 1–3, 2–3
Broadcast	1.80	1.11	1.86	1.12	1.80	1.09	1.75	1.11	1–2, 1–3, 2–3
Television	3.93	1.05	4.10	0.96	3.92	1.04	3.78	1.13	1–2, 1–3, 2–3
Internet	2.48	1.66	2.20	1.55	2.37	1.64	2.82	1.72	1–2, 1–3, 2–3
Cellphone	1.65	1.14	1.63	1.10	1.62	1.09	1.70	1.21	1–3, 2–3
Obesity (%)	6.5		5.7		6.2		7.6		1–3, 2–3
SHS	6.65	2.62	6.39	2.61	6.69	2.57	6.84	2.65	1–2, 1–3, 2–3

Note. N-Number, M-Mean, SD-Standard Deviation. Time change was tested by one-way analysis of variance (ANOVA) with Tamhane’s T2 since the unequal variances. 1–2 = significant difference in mean between 2013 and 2015, 1–3 = significant difference in mean between 2013 and 2015, 2–3 = significant difference in mean between 2013 and 2017. Detailed information is presented in supplementary material, Table S1.

**Table 2:** Logistic regression analysis of associations between media use and obesity in 2013, 2015, and 2017.

	2013			2015			2017		
	OR	95% CI		OR	95% CI		OR	95% CI	
Gender	0.98	0.83	1.16	0.95	0.81	1.11	0.96	0.84	1.10
Age	1.00	0.99	1.01	0.99 *	0.99	1.00	1.00	1.00	1.01
Rural/Urban	1.60 ***	1.32	1.94	1.52 ***	1.26	1.83	1.30 **	1.10	1.53
Illiterate	Reference N/A			Reference N/A			Reference N/A		
Primary	0.75 *	0.59	0.96	0.90	0.71	1.13	0.89	0.73	1.08
Middle school	0.73	0.51	1.04	0.62 *	0.43	0.90	0.80	0.61	1.06
High school	0.78	0.54	1.14	0.55 **	0.37	0.81	0.48 ***	0.35	0.66
College or Bachelor and above	0.58	0.20	1.62	0.41	0.15	1.15	0.31 **	0.13	0.72
Physical activity	1.02	0.96	1.09	1.04	0.98	1.10	1.02	0.98	1.07
Newspaper	1.06	0.97	1.16	0.97	0.87	1.07	0.95	0.87	1.03
Magazine	0.82 ***	0.73	0.91	0.96	0.85	1.08	1.00	0.90	1.11
Broadcast	1.07	1.00	1.16	1.09 *	1.01	1.18	0.99	0.92	1.05
Television	1.14 **	1.04	1.25	1.08 *	1.00	1.17	1.04	0.97	1.10
Internet	1.03	0.95	1.11	0.97	0.90	1.05	1.02	0.96	1.08
Cellphone	1.04	0.96	1.14	0.97	0.89	1.06	1.08 **	1.02	1.15
Constant	0.02 ***			0.05***			0.06 ***		

Note. Adjusted for gender, age, rural/urban, education, physical activity. * *p* < 0.05, ** *p* < 0.01, *** *p* < 0.001. N/A—not applicable. OR—odds ratio, 95% CI—95% confidence interval.

**Table 3:** Logistic regression analysis for the change in the associations between media use and obesity in 2013, 2015, and 2017.

	2013–2015 ^a^			2015–2017 ^b^			2013–2017 ^c^		
	OR	95% CI		OR	95% CI		OR	95% CI	
Gender	0.96	0.86	1.08	0.96	0.86	1.06	1.08	0.97	1.20
Age	1.00	0.99	1.00	1.00	0.99	1.00	1.00	1.00	1.01
Rural/Urban	1.56 ***	1.36	1.78	1.39 ***	1.23	1.57	1.15 *	1.02	1.30
Illiterate	Reference N/A			Reference N/A			Reference N/A		
Primary	0.82 *	0.69	0.97	0.89	0.76	1.03	0.94	0.80	1.10
Middle school	0.68 **	0.52	0.87	0.73 **	0.58	0.91	0.88	0.63	1.23
High school	0.66 **	0.51	0.86	0.50 ***	0.39	0.64	0.98	0.69	1.39
College or Bachelor and above	0.48 *	0.23	1.00	0.34 ***	0.18	0.65	0.74	0.26	2.06
Physical activity	1.03	0.99	1.08	1.03	0.99	1.07	1.03	0.99	1.07
Newspaper	1.06	0.97	1.16	0.97	0.88	1.07	1.09	0.99	1.19
Magazine	0.81 ***	0.73	0.91	0.97	0.86	1.09	0.80 ***	0.72	0.90
Broadcast	1.08 *	1.00	1.16	1.09*	1.01	1.17	1.09 *	1.01	1.17
Television	1.14 **	1.04	1.25	1.08	1.00	1.17	1.15 **	1.05	1.26
Internet	1.02	0.95	1.09	1.00	0.94	1.07	1.03	0.95	1.11
Cellphone	1.04	0.95	1.13	0.98	0.89	1.06	1.04	0.95	1.13
Year (Time period)	1.52	0.86	2.69	2.00 **	1.23	3.28	1.85 *	1.08	3.17
Newspaper*Year	0.90	0.79	1.03	0.98	0.87	1.12	0.84 **	0.73	0.95
Magazine*Year	1.18 *	1.00	1.39	1.02	0.87	1.20	1.34 ***	1.14	1.58
Broadcast*Year	1.00	0.90	1.11	0.91 *	0.82	1.00	0.92	0.83	1.02
Television*Year	0.95	0.84	1.08	0.96	0.87	1.07	0.93	0.83	1.04
Internet*Year	0.97	0.89	1.05	1.00	0.93	1.07	0.98	0.90	1.07
Cellphone*Year	0.94	0.83	1.06	1.11 *	1.00	1.23	1.05	0.94	1.18
Constant	0.02 ***			0.04 ***			0.02 ***		

Note. Adjusted for gender, age, rural/urban, education, physical activity, * *p* < 0.05, ** *p* < 0.01, *** *p* < 0.001. OR—odds ratio, 95% CI—95% confidence interval. ^a^: The pairwise time points are dummy-coded as the independent variable “Year”, 2013 was coded “0”and 2015 was coded “1”. ^b^: The pairwise time points are dummy-coded as the independent variable “Year”, 2015 was coded “0”and 2017 was coded “1”. ^c^: The pairwise time points are dummy-coded as the independent variable “Year”, 2013 was coded “0”and 2017 was coded “1”.

**Table 4:** Hierarchical multiple regression analysis of associations between media use and SHS in 2013, 2015, and 2017.

	2013		2015		2017	
	Model1 Model2		Model1 Model2		Model1 Model2	
	Beta	Beta	Beta	Beta	Beta	Beta
Gender	0.05 ***	0.05 ***	0.07 ***	0.06 ***	0.06 ***	0.06 ***
Age	0.33 ***	0.30 ***	0.32 ***	0.27 ***	0.30 ***	0.24 ***
Rural/Urban	−0.06 ***	−0.04 ***	−0.07 ***	−0.04 ***	−0.11 ***	−0.07 ***
Illiterate	Reference N/A		Reference N/A		Reference N/A	
Primary	−0.05 ***	−0.03 *	−0.06 ***	−0.03 **	−0.06 ***	−0.03 ***
Middle school	−0.03 ***	−0.01	−0.06 ***	−0.03 **	−0.06 ***	−0.03 ***
High school	−0.03 **	−0.01	−0.03 ***	0.01	−0.04 ***	−0.02 *
College or Bachelor and above	−0.01	−0.01	−0.02	−0.01	−0.01	0.01
Obesity	0.01	0.01	0.03 ***	0.03 ***	0.04 ***	0.04 ***
Physical activity	−0.09 **	−0.07 ***	−0.11 ***	−0.07 ***	−0.15 ***	−0.12 ***
Newspaper		−0.06 ***		−0.06 ***		−0.07 ***
Magazine		0.01		−0.01		0.03 *
Broadcast		0.05 ***		0.01		0.01
Television		−0.12 ***		−0.10 ***		−0.06 ***
Internet		−0.08 ***		−0.13 ***		−0.16 ***
Cellphone		0.01		0.01		0.01
Adjusted R2	0.156	0.178	0.163	0.183	0.188	0.206
R2 change	0.156	0.023	0.164	0.020	0.189	0.018
F	231.569 ***	52.839 ***	235.457 ***	44.619 ***	319.696 ***	45.924 ***

Note. Adjusted for gender, age, rural/urban, education, physical activity, and obesity. * *p* < 0.05, ** *p* < 0.01, *** *p* < 0.001. N/A—not applicable.

**Table 5:** Hierarchical multiple regression analysis for the change in associations between media use and SHS in 2013, 2015, and 2017.

	2013–2015 ^a^			2015–2017 ^b^			2013–2017 ^c^		
	Model1 Model2 Model3			Model1 Model2 Model3			Model1 Model2 Model3		
	Beta	Beta	Beta	Beta	Beta	Beta	Beta	Beta	Beta
Gender	0.06 ***	0.05 ***	0.05 ***	0.06 ***	−0.06 ***	0.06 ***	0.06 ***	0.05 ***	0.05 ***
Age	0.33 ***	0.28 ***	0.28 ***	0.30 ***	0.25 ***	0.25 ***	0.32 ***	0.27 ***	0.27 ***
Rural/Urban	−0.07 ***	−0.04 ***	−0.04 ***	−0.08 ***	−0.08 ***	−0.08 ***	−0.12 ***	−0.08 ***	−0.08 ***
Illiterate	Reference N/A			Reference N/A			Reference N/A		
Primary	−0.06 ***	−0.03 ***	−0.03 ***	−0.07 ***	−0.03 ***	−0.03 ***	−0.06 ***	−0.03 ***	−0.03 ***
Middle school	−0.04 ***	−0.02 **	−0.02 **	−0.07 ***	−0.03 ***	−0.03 ***	−0.05 ***	−0.02 ***	−0.02 ***
High school	−0.03 ***	−0.01	−0.01	−0.05 ***	−0.01 *	−0.01	−0.03	−0.02 *	−0.01 *
College or Bachelor and above	−0.01	−0.01	−0.01	−0.02 **	−0.01	−0.01	−0.01	0.01	0.01
Obesity	0.02 **	0.02 **	0.02 **	0.03 ***	0.03 ***	0.03 ***	0.02 ***	0.02 ***	0.02 ***
Physical activity	−0.09 ***	−0.07 ***	−0.07 ***	−0.14 ***	−0.10 ***	−0.10 ***	−0.13 ***	−0.10 ***	−0.10 ***
Newspaper		−0.06 ***	−0.06 ***		−0.06 ***	−0.04 ***		−0.07 ***	−0.05 ***
Magazine		0.01	0.01		0.01	−0.01		0.01	0.01
Broadcast		0.03 ***	0.05 ***		0.01	0.02 *		0.02 ***	0.05 ***
Television		−0.11 ***	−0.13 ***		−0.08 ***	−0.10 ***		−0.09 ***	−0.13 ***
Internet		−0.11 ***	−0.10 ***		−0.15 ***	−0.13 ***		−0.12 ***	−0.10 ***
Cellphone		0.01	0.01		0.01	0.01		0.01	0.01
Year (Time period)		0.04 ***	0.01		−0.02	−0.03		0.03 **	−0.02
Newspaper*Year			0.01			−0.04 *			−0.04 ***
Magazine*Year			0.01			0.03			0.03
Broadcast*Year			−0.04 **			−0.03			−0.07 ***
Television*Year			0.07 **			0.08 ***			0.15 ***
Internet*Year			−0.02			−0.03 *			−0.05 **
Cellphone*Year			0.01			−0.01			−0.01
Adjusted R2	0.160	0.183	0.183	0.175	0.194	0.195	0.177	0.195	0.198
R2 change	0.160	0.023	0.001	0.175	0.020	0.001	0.77	0.018	0.003
F	467.193 ***	89.975 ***	3.276 **	547.582 ***	80.405 ***	5.116 ***	565.152 ***	73.536 ***	15.976 ***

Note. Adjusted for gender, age, rural/urban, education, physical activity, and obesity. * *p* < 0.05, ** *p* < 0.01, *** *p* < 0.001. N/A—not applicable. ^a^: The pairwise time points are dummy-coded as the independent variable “Year”, 2013 was coded “0”, and 2015 was coded “1”. ^b^: The pairwise time points are dummy-coded as the independent variable “Year”, 2015 was coded “0”, and 2017 was coded “1”. ^c^: The pairwise time points are dummy-coded as the independent variable “Year”, 2013 was coded “0”, and 2017 was coded “1”.

## Data Availability

Publicly available datasets were analyzed in this study. This data can be found here: http://cnsda.ruc.edu.cn/index.php?r=site/article&id=180.

## Supplementary Materials

The following are available online at https://www.mdpi.com/article/10.3390/ijerph182413214/s1, Table S1. The difference of media use, obesity and SHS among 2013, 2015, and 2017(ANOVA).
