# Supplementary material for: The Interactions of Media Use, Obesity, and Suboptimal Health Status: A Nationwide Time-Trend Study in China
Source: Int J Environ Res Public Health. 2021 Dec 15;18(24):13214. doi: 10.3390/ijerph182413214 (PMC8701945; doi:10.3390/ijerph182413214)
Supplement: Supplementary file 1 [file ijerph-18-13214-s001.zip › ijerph-1457477-supplementary.pdf]

Table S1. The difference of media use, obesity and SHS among 2013, 2015, and 2017(ANOVA).

**Post Hoc Multiple Comparison**

Tamhane's T2

| Dependent | Year I | Year J | Difference Vale of Mean | Standard Error | p | 95%CI | |
|---|---|---|---|---|---|---|---|
| Newspaper | 1.00 | 2.00 | .201* | 0.016 | < 0.001 | 0.16 | 0.24 |
| | | 3.00 | .330* | 0.015 | < 0.001 | 0.29 | 0.37 |
| | 2.00 | 1.00 | -.201* | 0.016 | < 0.001 | -0.24 | -0.16 |
| | | 3.00 | .129* | 0.015 | < 0.001 | 0.09 | 0.16 |
| | 3.00 | 1.00 | -.330* | 0.015 | < 0.001 | -0.37 | -0.29 |
| | | 2.00 | -.129* | 0.015 | < 0.001 | -0.16 | -0.09 |
| Magazine | 1.00 | 2.00 | .109* | 0.013 | < 0.001 | 0.08 | 0.14 |
| | | 3.00 | .220* | 0.012 | < 0.001 | 0.19 | 0.25 |
| | 2.00 | 1.00 | -.109* | 0.013 | < 0.001 | -0.14 | -0.08 |
| | | 3.00 | .112* | 0.012 | < 0.001 | 0.08 | 0.14 |
| | 3.00 | 1.00 | -.220* | 0.012 | < 0.001 | -0.25 | -0.19 |
| | | 2.00 | -.112* | 0.012 | < 0.001 | -0.14 | -0.08 |
| Broadcast | 1.00 | 2.00 | .059* | 0.015 | < 0.001 | 0.02 | 0.09 |
| | | 3.00 | .113* | 0.015 | < 0.001 | 0.08 | 0.15 |
| | 2.00 | 1.00 | -.059* | 0.015 | < 0.001 | -0.09 | -0.02 |
| | | 3.00 | .054* | 0.014 | 0.001 | 0.02 | 0.09 |
| | 3.00 | 1.00 | -.113* | 0.015 | < 0.001 | -0.15 | -0.08 |
| | | 2.00 | -.054* | 0.014 | 0.001 | -0.09 | -0.02 |
| Television | 1.00 | 2.00 | .171* | 0.013 | < 0.001 | 0.14 | 0.20 |
| | | 3.00 | .311* | 0.014 | < 0.001 | 0.28 | 0.34 |
| | 2.00 | 1.00 | -.171* | 0.013 | < 0.001 | -0.20 | -0.14 |
| | | 3.00 | .140* | 0.014 | < 0.001 | 0.11 | 0.17 |
| | 3.00 | 1.00 | -.311* | 0.014 | < 0.001 | -0.34 | -0.28 |
| | | 2.00 | -.140* | 0.014 | < 0.001 | -0.17 | -0.11 |
| Internet | 1.00 | 2.00 | -.171* | 0.021 | < 0.001 | -0.22 | -0.12 |
| | | 3.00 | -.616* | 0.021 | < 0.001 | -0.67 | -0.56 |
| | 2.00 | 1.00 | .171* | 0.021 | < 0.001 | 0.12 | 0.22 |
| | | 3.00 | -.445* | 0.022 | < 0.001 | -0.50 | -0.39 |
| | 3.00 | 1.00 | .616* | 0.021 | < 0.001 | 0.56 | 0.67 |
| | | 2.00 | .445* | 0.022 | < 0.001 | 0.39 | 0.50 |
| Cellphone | 1.00 | 2.00 | 0.010 | 0.015 | 0.881 | -0.03 | 0.04 |
| | | 3.00 | -.069* | 0.015 | < 0.001 | -0.11 | -0.03 |
| | 2.00 | 1.00 | -0.010 | 0.015 | 0.881 | -0.04 | 0.03 |
| | | 3.00 | -.079* | 0.015 | < 0.001 | -0.12 | -0.04 |
| | 3.00 | 1.00 | .069* | 0.015 | < 0.001 | 0.03 | 0.11 |
| | | 2.00 | .079* | 0.015 | < 0.001 | 0.04 | 0.12 |
| Obesity | 1.00 | 2.00 | -0.00509 | 0.00318 | 0.294 | -0.01 | 0.01 |
| | | 3.00 | -.01886* | 0.00323 | < 0.001 | -0.03 | -0.01 |

| | | | | | | | |
|---|---|---|---|---|---|---|---|
| | 2.00 | 1.00 | 0.00509 | 0.00318 | 0.294 | -0.01 | 0.01 |
| | | 3.00 | -.01377* | 0.00332 | < 0.001 | -0.02 | -0.01 |
| | 3.00 | 1.00 | .01886* | 0.00323 | < 0.001 | 0.01 | 0.03 |
| | | 2.00 | .01377* | 0.00332 | < 0.001 | 0.01 | 0.02 |
| SHS | 1.00 | 2.00 | -.10253* | 0.01162 | < 0.001 | -0.13 | -0.07 |
| | | 3.00 | -.15204* | 0.01142 | < 0.001 | -0.18 | -0.12 |
| | 2.00 | 1.00 | .10253* | 0.01162 | < 0.001 | 0.07 | 0.13 |
| | | 3.00 | -.04951* | 0.01143 | < 0.001 | -0.08 | -0.02 |
| | 3.00 | 1.00 | .15204* | 0.01142 | < 0.001 | 0.12 | 0.18 |
| | | 2.00 | .04951* | 0.01143 | < 0.001 | 0.02 | 0.08 |

Not. *: Significant level of difference value of mean is 0.05.

SHS: Suboptimal Health Status: A Nationwide
